# Implication of epithelial-mesenchymal transition in IGF1R-induced resistance to EGFR-TKIs in advanced non-small cell lung cancer

**DOI:** 10.18632/oncotarget.6293

**Published:** 2015-11-05

**Authors:** Juan Zhou, Jinjing Wang, Yunyun Zeng, Xi Zhang, Qiaoting Hu, Jihua Zheng, Bei Chen, Bo Xie, Wei-Min Zhang

**Affiliations:** ^1^ Department of Oncology, Guangzhou Clinical College of the Second Military Medical University, Guangzhou, Guangdong 510010, China; ^2^ Department of Oncology, General Hospital of Guangzhou Military Command of PLA, Guangzhou, Guangdong 510010, China

**Keywords:** epidermal growth factor receptor-tyrosine kinase inhibitors, epithelial-mesenchymal transition, type 1 insulin-like growth factor receptor, non-small cell lung cancer, drug resistance

## Abstract

The underlying mechanisms for acquired resistance to epidermal growth factor receptor-tyrosine kinase inhibitors (EGFR-TKIs) in about 30%-40% of non-small cell lung cancer (NSCLC) patients remain elusive. Recent studies have suggested that activation of epithelial-mesenchymal transition (EMT) and type 1 insulin-like growth factor receptor (IGF1R) is associated with acquired EGFR-TKIs resistance in NSCLC. Our study aims to further explore the mechanism of EMT and IGF1R in acquired EGFR-TKIs resistance in NSCLC cell lines with mutant (PC-9) or wild-type EGFR (H460). Compared to parental cells, EGFR-TKIs-resistant PC-9/GR and H460/ER cells displayed an EMT phenotype and showed overexpression of IGF1R. SiIGF1R in PC-9/GR and H460/ER cells reversed EMT-related morphologies and reversed their resistance to EGFR-TKIs. Exogenous IGF-1 alone induced EMT in EGFR-TKIs-naïve PC-9 and H460 cells and increased their resistance against EGFR-TKIs. Inducing EMT by TGF-β1 in PC-9 and H460 cells decreased their sensitivity to EGFR-TKIs, whereas reversing EMT by E-cadherin overexpression in PC-9/GR and H460/ER cells restored their sensitivity to EGFR-TKIs. These data suggest that IGF1R plays an important role in acquired drug resistance against EGFR-TKIs by inducing EMT. Targeting IGF1R and EMT may be a potential therapeutic strategy for advanced NSCLC with acquired EGFR-TKIs resistance.

## INTRODUCTION

Acquired drug resistance has become a bottleneck in the treatment of advanced non-small cell lung cancer (NSCLC) using epidermal growth factor receptor-tyrosine kinase inhibitors (EGFR-TKIs) [1–3]. Currently, the known mechanisms underlying this acquired drug resistance include T790M mutation [4, 5] and amplification of *MET* genes [6]. These mechanisms account for about 60–70% of acquired drug resistance. However, the underlying mechanisms for approximately 30%-40% of cases are still unclear. Recent studies show that the activation of epithelial-mesenchymal transition (EMT) and type 1 insulin-like growth factor receptor (IGF1R) is associated with acquired drug resistance against EGFR-TKIs in NSCLC [7, 8].

The insulin-like growth factor (IGF) system, including IGF ligands, their receptors and binding proteins, is important in promoting tumor development. Previous studies showed that activation of IGF1R is involved in EGFR-TKIs resistance in NSCLC cell lines [9] and in lung cancer patients [10]. IGF1R tyrosine kinase inhibitors have been reported to reverse the drug resistance of NSCLC to EGFR-TKIs *in vitro* and *in vivo* [7]. IGF1R activates the downstream pathways of EGFR signaling, such as the phosphatidylinositol 3 kinase/protein kinase B (PI3K/AKT) pathway and the extracellular signal-regulated kinases/mitogen-activated protein kinase (ERK/MAPK) pathway, leading to secondary drug resistance to EGFR-TKIs [11–13]. However, the exact mechanisms of IGF1R-induced acquired EGFR-TKIs resistance remain to be elucidated.

Interestingly, IGF1R has been shown to play an important role in EMT [7] and IGF1R activation can induce EMT in breast epithelial cells [14] and prostate cancer cells [15]. EMT is a biological process of losing epithelial features and acquiring mesenchymal properties, characterized by E-cadherin reduction and Vimentin induction. It has been reported that a subgroup of NSCLC with pronounced EMT was EGFR-TKIs resistant [3, 8, 16, 17], suggesting that EMT may render NSCLC insensitive to EGFR inhibition. Furthermore, decreased expression of E-cadherin [8, 16, 17] was associated with reduced sensitivity to EGFR-TKIs, and restoration of E-cadherin expression improved cells' sensitivity to EGFR-TKIs [18]. Consistently, clinical studies have suggested a prognostic value of E-cadherin in NSCLC patients treated with EGFR-TKIs [19–21].

Previously, we reported the association between EMT, IGF1R expression and drug response in advanced NSCLC patients treated with gefitinib [22]. NSCLC patients with negative EMT or lower IGF1R expression have a significantly higher objective response rate. Both, IGF1R expression and EMT occurrence correlated with the development of acquired drug resistance to EGFR-TKIs in NSCLC patients. In the present study, we further examined the relationship between EMT and IGF1R expression with sensitivity to EGFR-TKIs in NSCLC cell lines with wild-type or mutant *EGFR*. Furthermore, using *in vitro* assays, we provided evidence that IGF1R induced EGFR-TKIs resistance by inducing EMT and explored the possible cellular mechanism. Our data highlight the importance of EMT in IGF1R-induced resistance to EGFR-TKIs in NSCLC and implicate both EMT and IGF1R as potential therapeutic targets for advanced NSCLC.

## RESULTS

### IGF1R activation is involved in the acquirement of the EGFR-TKIs-resistance phenotype

**Figure 1:**
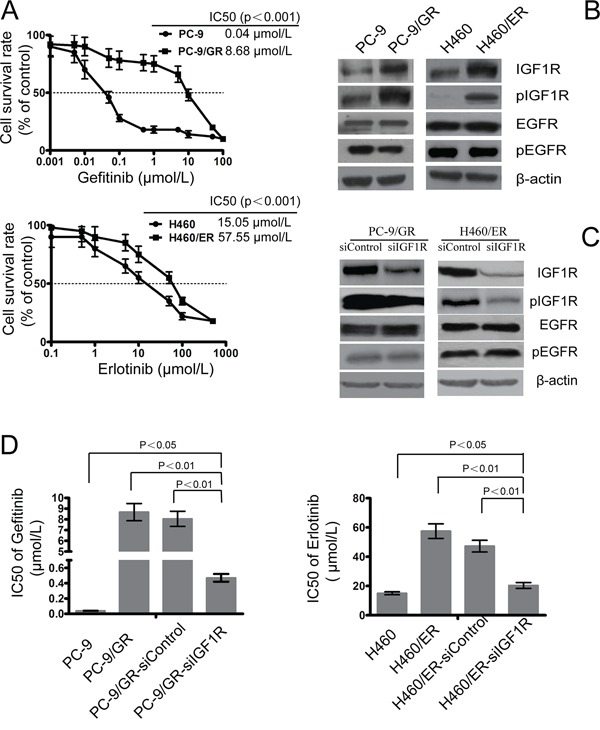
Role of IGF1R on the sensitivity to gefitinib and erlotinib in EGFR-TKIs-resistant cells **A.** The sensitivity to gefitinib and erlotinib of PC-9/GR, H460/ER, and their parental cells was assessed by MTT assays. Cells were treated with the indicated doses of gefitinib or erlotinib for 72 h. IC50 values for different conditions are provided in the table within individual figures. **B.** Expression of IGF1R, phosphor-IGF1R, EGFR, and phosphor-EGFR in EGFR-TKIs-resistant cells by immunoblotting analysis. **C.** Effect of IGF1R siRNA on expression of IGF1R, phosphor-IGF1R, EGFR, and phosphor-EGFR in EGFR-TKIs-resistant cells. β-actin was used as an internal control. **D.** IC50 of gefitinib/erlotinib in PC-9/GR and H460/ER cells increased significantly following IGF1R knock-down when compared with the control cells. Data represent means ± S.D. of three independent experiments.

As expected, the resistant cells PC-9/GR and H460/ER exhibited significantly decreased sensitivity to EGFR-TKIs, compared to the parental PC-9 and H460 cells, respectively (Figure 1A). The delE746-A750 deletion mutation in exon 19 of EGFR was detected in PC-9 and PC-9/GR cells by qPCR-HRM, but not in H460 or H460/ER; however, the T790M mutation was not detected in any of the cell lines. FISH analysis showed no amplification of *c-MET* in PC-9/GR or H460/ER cells (Supplementary Figure S2). No *EGFR* mutation in H460/ER cells was found, and all cell lines harbored wild-type *KRAS* before and after the induction of drug resistance (Supplementary Figure S3). Additionally, the expression of IGF1R and the phosphorylation of IGF1R (pIGF1R) increased significantly in PC-9/GR and H460/ER cells after the acquisition of drug resistance, while the expression of EGFR and the phosphorylation of EGFR (pEGFR) showed no significant changes (Figure 1B).

To further evaluate whether activation of the IGF1R pathway was the major mechanism underlying acquired drug resistance, we targeted silencing of IGF1R using siRNA technology in PC-9/GR and H460/ER cells. The expression of IGF1R in PC-9/GR-siIGF1R and H460/ER-siIGF1R cells was downregulated, indicating that the interference was successful, while the expression of EGFR and pEGFR did not show significant changes (Figure 1C). PC-9/GR-siIGF1R and H460/ER-siIGF1R cells had the same *EGFR* and *KRAS* gene state as their parent cells (Supplementary Figure S4). Furthermore, knockdown of IGFIR significantly decreased the IC50 of gefitinib and erlotinib in PC-9/GR and H460/ER cells, respectively, suggesting that IGFIR may play an important role in restoring their sensitivity to gefitinib or erlotinib (Figure 1D).

### EMT is involved in the acquirement of the EGFR-TKIs-resistance phenotype

**Figure 2:**
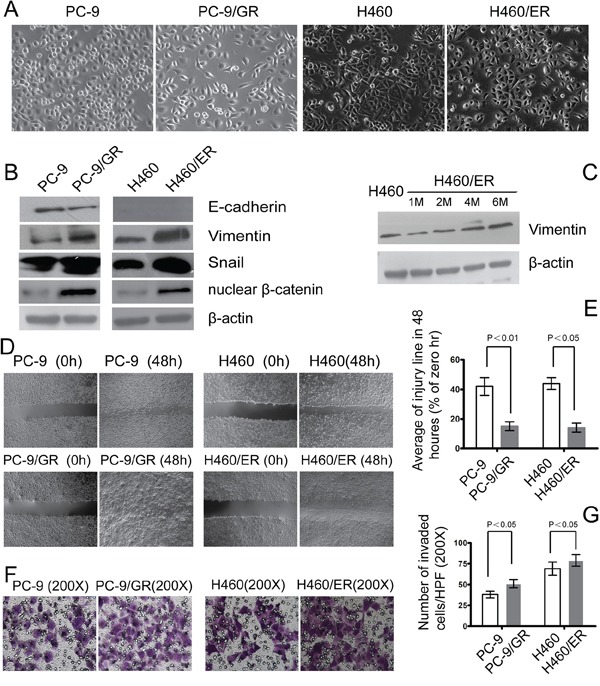
EMT in EGFR-TKIs-resistant cells **A.** Morphology of PC-9/GR, H460/ER, and their parental cells grown for 3 days until 90% confluence. In contrast to the parental cells, the PC-9/GR and H460/ER cells displayed long spindle-like shape with loose cell junctions. Photographs were taken at × 200 magnification. **B–C.** Loss of E-cadherin was seen in PC-9/GR cells, H460 cells did not express E-cadherin, and there was increased expression of Vimentin, transcription factor Snail and nuclear β-catenin in PC-9/GR and H460/ER cells shown by immunoblotting analysis. In addition, Vimentin expression at the protein level increased in a time dependent manner after the induction of drug resistance. β-actin was used as an internal control. **D, E.** Enhanced migratory capacity of EGFR-TKIs-resistant cells according to Scratch assay. Confluent cells were scraped by a pipette tip to generate wounds and then were cultured in serum-free culture medium for 48 h. Representative images of wounds were taken at 0 and 48 h. Cell motility was examined with a light microscope (×40) and the width of the wound was quantified. **F, G.** Enhanced invasiveness of EGFR-TKIs-resistant cells according to transwell assay. The cells were incubated for 24 h in modified Boyden chambers. Those cells that migrated through the filters were stained and counted under a light microscope. Quantification was done in 10 randomly chosen fields. The data are reported as means ± S.D. The photographs were taken at × 200 magnification.

After the acquisition of drug resistance, PC-9/GR and H460/ER cells showed EMT phenotypes, with loose cell junctions and long, spindle-type morphology (Figure 2A). Expression of the epithelial marker E-cadherin decreased in PC-9/GR cells, and was undetectable in H460 and H460/ER cells. In contrast, the mesenchymal marker Vimentin, transcription factor Snail and nuclear β-catenin all increased in PC-9/GR and H460/ER cells, compared to their parental cells (Figure 2B). Specifically, Vimentin expression gradually increased in H460/ER cells in a time-dependent manner upon erlotinib incubation (Figure 2C). Cell migration and invasion abilities increased significantly in PC-9/GR and H460/ER cells compared to their parental cells as shown by scratch test and transwell experiment (Figure 2D, 2E, 2F, 2G).

**Figure 3:**
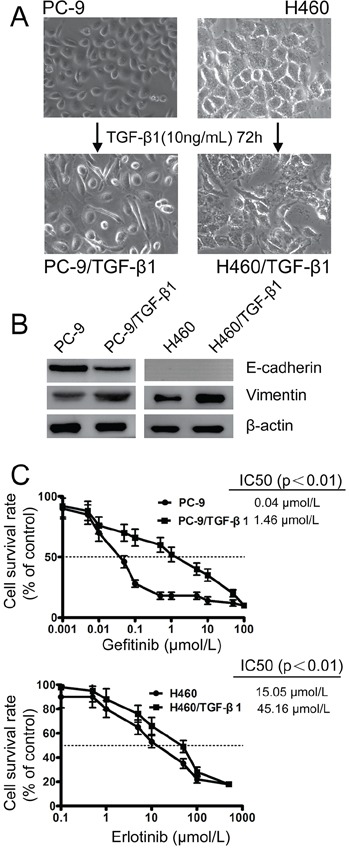
Effect of TGF-β1 on EMT and the sensitivity to gefitinib and erlotinib in EGFR-TKIs-resistant cells **A.** Morphology of PC-9 and H460 cells grown with 10 ng/mL TGF-β1 for 3 days until 90% confluence. The photographs were taken at × 200 magnification. **B.** TGF-β1-induced downregulation of E-cadherin and upregulation of Vimentin in PC-9 and H460 cells according to immunoblotting analysis. β-actin was used as an internal control. **C.** The effects of sequential treatment with the TGF-β1 on cell viability of PC-9 and H460 cells exposed to gefitinib and erlotinib by MTT uptake assays, respectively. The data represent the means ± S.D. of three independent experiments.

Treatment with TGF-β1 has also been shown in many cancer cells to promote a shift from epithelial to mesenchymal phenotype [25, 26]. To further evaluate whether EMT constituted a major mechanism underlying acquired drug resistance, parental PC-9 and H460 cells were treated with exogenous TGF-β1 (10 ng/mL) for 72 h. After TGF-β1 treatment, both PC-9 and H460 cells acquired a spindle-like morphology (Figure 3A) and expressed notably reduced E-cadherin and increased Vimentin proteins (Figure 3B). Interestingly, following TGF-β1-induced transient EMT, the sensitivity of PC-9 and H460 to EGFR-TKIs was dramatically reduced (Figure 3C), which phenocopied the PC-9/GR and H460/ER cells, respectively. These results indicated that transient induction of mesenchymal-like phenotypes is sufficient to induce resistance to EGFR-TKIs in NSCLC cells.

### Inhibition of EMT increased sensitivity to EGFR-TKIs in NSCLC cells

**Figure 4:**
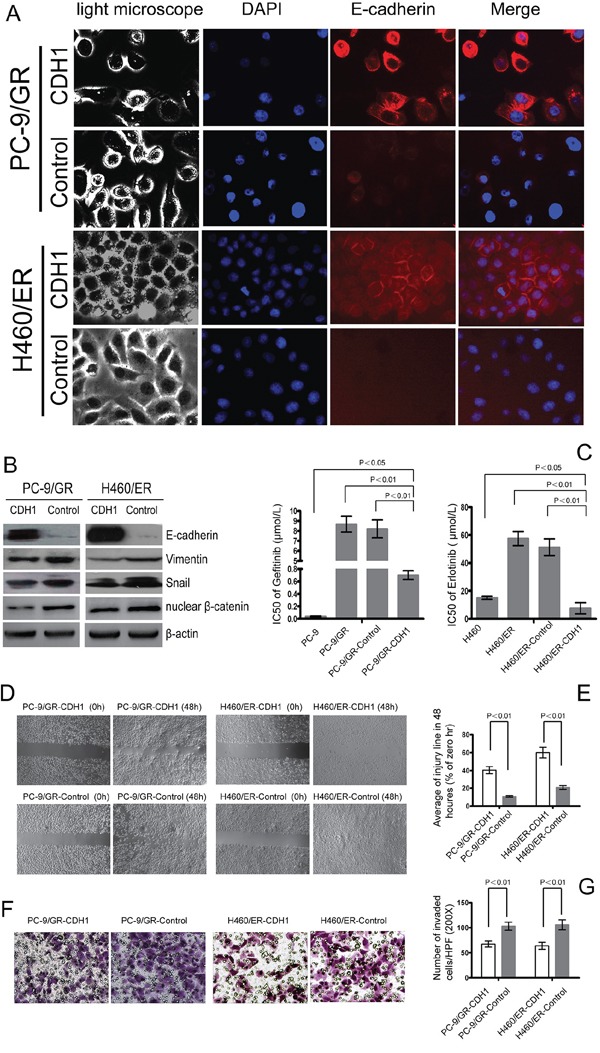
Effect of E-cadherin (CDH1) overexpression on EMT and the sensitivity to gefitinib and erlotinib in EGFR-TKIs-resistant cells **A.** E-cadherin-overexpressing cell lines PC-9/GR and H460/ER after CDH1 transfection showed an epithelial-like morphology and a remarkably increased expression of E-cadherin according to immunofluorescence assay. The nuclei were stained with DAPI (blue fluorescence), and E-cadherin was stained with Cy3-conjugated antibodies (red fluorescence). **B.** Effect of E-cadherin overexpression on expression levels of EMT markers in EGFR-TKIs resistant cells. β-actin was used as an internal control. **C.** The IC50 of gefitinib/erlotinib in E-cadherin-overexpressing PC-9/GR-CDH1 and H460/ER-CDH1 cells was significantly greater than that in PC-9/GR-control and H460/ER-control cells. The data represent the means ± S.D. of three independent experiments. **D, E.** E-cadherin overexpression repressed migration of EGFR-TKIs-resistant cells according to Scratch assay. **F, G.** E-cadherin overexpression suppressed invasion of EGFR-TKIs-resistant cells according to transwell assay.

To further evaluate the role of EMT in the resistance to EGFR-TKIs in NSCLC cells, *CDH1* which encodes E-cadherin was stably transfected into PC-9/GR and H460/ER cells. The *EGFR* and *KRAS* gene status in the resulting cells, PC9/GR-CDH1 and H460/ER-CDH1, remained the same as their parent cells (Supplementary Figure S5). PC9/GR-CDH1 and H460/ER-CDH1 presented an epithelial-like morphology (Figure 4A). The expression of E-cadherin in PC-9/GR-CDH1 and H460/ER-CDH1 cells was significantly higher than that in the empty vector controls, indicating that the lentiviral transfection of E-cadherin was successful. Additionally, compared to the empty vector control, the expression of Vimentin, Snail and nuclear β-catenin decreased in PC-9/GR-CDH1 and H460/ER-CDH1 cells (Figure 4B). Overexpression of CDH1 correlated with a reduction of the nuclear β-catenin in PC-9/GR and H460/ER by immunofluorescence staining experiment (Supplementary Figure S6A). Most importantly, the sensitivity to gefitinib and erlotinib dramatically increased in E-cadherin-overexpressing PC9/GR and H460/ER cells, respectively (Figure 4C), suggesting that E-cadherin repressed EMT in NSCLC cells, and subsequently enhanced the cytotoxic effect of EGFR-TKIs. Finally, overexpression of E-cadherin inhibited the motility and invasiveness of EGFR-TKIs-resistant NSCLC cells shown by scratch test and transwell assays (Figure 4D, 4E, 4F, 4G).

### Exogenous IGF-1 induced EMT in EGFR-TKIs-naïve NSCLC cells and increased their resistance to EGFR-TKIs

**Figure 5:**
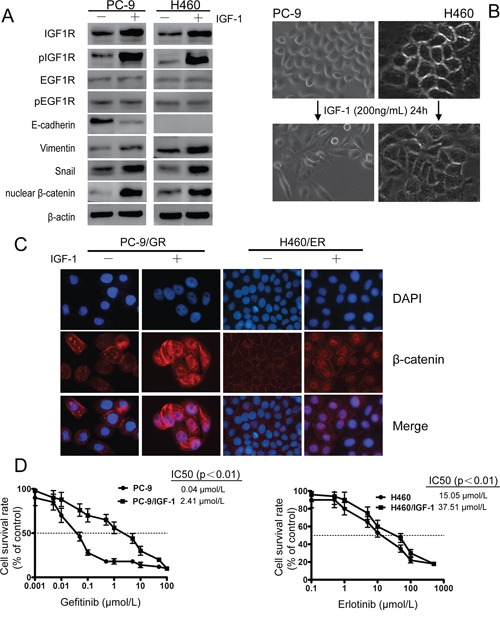
IGF1R activation led to EMT and decreased sensitivity against EGFR-TKIs in PC-9 and H460 cells upon IGF-1 induction EGFR-TKIs-naïve lung cancer cells PC-9 and H460 were serum-starved overnight and then treated with fresh RPMI 1640 containing 0.5% FBS and 200 ng/ml IGF-I for 24 h. **A.** After IGF-1 induction, IGF1R and pIGF1R were activated. EMT phenotype, decreased expression of E-cadherin, increased Vimentin, nuclear β-catenin and Snail were observed. β-actin was used as an internal control. **B.** Mesenchymal phenotype of PC-9 and H460 cells after IGF-1 induction. **C.** β-catenin relocated from cell membrane to nucleus after IGF-1 induction as shown by immunofluorescence experiment. The photographs were taken at × 200 magnification. **D.** Exogenous IGF-1 application increased resistance to EGFR-TKIs in PC-9 and H460 cells. Data represent means ± S.D.of three independent experiments.

To test whether IGF1R overexpression alone can trigger EMT and EGFR-TKIs resistance, exogenous IGF-1 was applied to EGFR-TKIs-naïve PC-9 and H460 cells. Twenty-four hours after IGF-1 induction, both IGF1R and pIGF1R were induced, and no significant change of EGFR was observed (Figure 5A). The IGF-1 induced cells also acquired a mesenchymal phenotype and a spindle-like morphology (Figure 5B). EMT occurrence was further confirmed by decreased expression of E-cadherin, and increased expression of Vimentin, nuclear β-catenin and Snail (Figure 5A). In addition, β-catenin was shown to translocate from cell membrane into the nuclear after IGF-1 induction by Immunofluorescence experiment (Figure 5C). IGF-1 alone does not promote lung cancer cell proliferation (data not shown), however IGF-1 induced PC-9 and H460 cells showed decreased sensitivity to EGFR-TKIs compared to parental cells (Figure 5D). These results indicated that activation of IGF1R is associated with EMT process.

### IGF1R induced EMT of NSCLC cells by enhancing ERK/MAPK signaling

**Figure 6:**
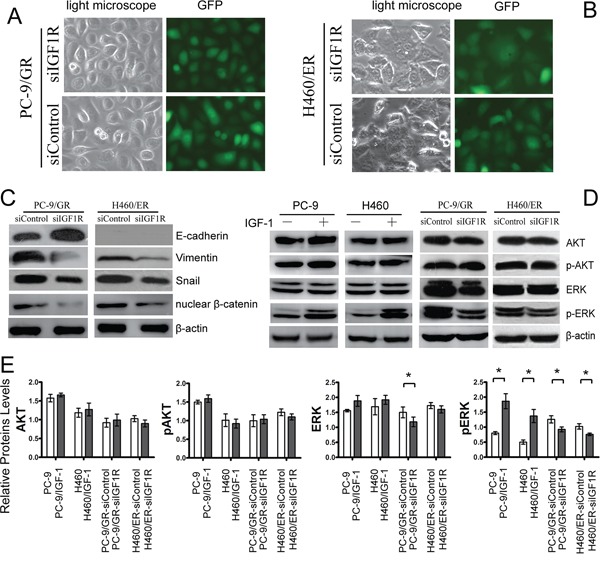
Effects of IGF1R on EMT and ERK/MAPK signaling of EGFR-TKIs-resistant cells **A, B.** Morphologic change of PC-9/GR and H460/ER after IGF1R knockdown. Green fluorescence represented the successful transfection of IGF1R siRNA. **C.** Upregulation of E-cadherin. and downregulation of Vimentin. nuclear β-catenin and Snail by IGF1R silencing. **D, E.** Attenuated ERK/MAPK signaling in PC-9 and H460 after IGF1R activation by IGF-1 and IGF1R knockdown. β-actin was used as an internal control.* *p* < 0.05

To clarify the association among IGF1R activation, EMT, and resistance to EGFR-TKIs, we explored whether the induction of EMT might proceed through the activation of IGF1R to promote resistance to EGFR-TKIs in NSCLC cells. Knockdown of IGF1R by siRNA repressed the EMT-related morphological features (Figure 6A and 6B). We also demonstrated that knockdown of IGF1R attenuated the decrease of E-cadherin expression and the increase of Vimentin, Snail and nuclear β-catenin expression in PC9/GR and H460/ER cells (Figure 6C, Supplementary Figure S6B). Furthermore, activation of IGF1R by IGF-1 induced activation of pERK, but not of pAKT in PC9 and H460 cells (Figure 6D and 6E). Finally, knockdown of IGF1R dramatically attenuated the increase in total ERK and pERK in PC-9/GR, and resulted in decreased pERK in H460/ER (Figure 6D and 6E).

## DISCUSSION

In the present study, both PC-9 cells, which harbor the mutant EGFR, and H460 cells, with wild-type EGFR, were used to establish EGFR-TKIs-resistant sublines (PC-9/GR and H460/ER) by continuously culturing in gefitinib or erlotinib, respectively. We did not detect the classical T790M mutation or *c-MET* gene amplification in either PC-9/GR or H460/ER cells. However, expression of IGF1R and pIGF1R, but not that of EGFR, were enhanced in these two resistant cell lines. These results suggested that IGF1R has a major role in acquired drug resistance to EGFR-TKIs, which is consistent with previous studies. For example, Morgillo *et al*. [7] found decreased EGFR and increased pIGF1R in erlotinib-resistant H460 cells. They also found that the combined treatment of an IGF1R tyrosine kinase inhibitor with erlotinib inhibits cell proliferation significantly. Vazquez-Martin *et al*. [27] and Cortot *et al*. [28] also suggested a role of IGF1R in acquired drug resistance to EGFR-TKIs in PC-9 cells.

To further evaluate the role of IGF1R in acquired EGFR-TKIs resistance, we knocked down IGF1R by siRNA in PC-9/GR and H460/ER cells and found that both cells restored their sensitivity to gefitinib or erlotinib. Furthermore, our data showed that exogenous IGF-1 application in EGFR-TKIs-naïve PC-9 and H460 cells triggered IGF1R activation, and increased cells' resistance to EGFR-TKIs. Guix *et al.* [29] also discovered that IGF-1 activated IGF1R in lung cancer cells, and showed that only cells, which depended on IGF1R and its downstream signaling pathways to promote cell growth, acquired the resistance ability against geftinib. Taken together, our results provided direct evidence that activation of IGF1R is one of the mechanisms underlying acquired drug resistance to EGFR-TKIs in lung cancer cells.

There are still few studies addressing the mechanism of IGF1R activation in EGFR-TKIs resistance in lung cancer cells. One possible mechanism is that after EGFR pathway is inhibited, IGF1R pathway is activated to promote cell survival and proliferation [7]. Morgillo *et al.* [7] found that erlotinib treatment of H460 cells increase the levels of EGFR/IGFIR heterodimer on cell membrane, subsequently activate IGFIR and its downstream signaling mediators. Data in the present study, which were consistent with Peled *et al*'s [9] report, showed that exogenous IGF-1 alone does not promote lung cancer cell proliferation, but instead increases cell survival against erlotinib. Further study is needed in this regard.

It is well established that EMT is involved in acquired drug resistance to EGFR-TKIs in NSCLC cells carrying wild-type EGFR, for instance, in A549 [8, 30] and in H1229 cells [30]. However, there is still paucity of evidence to support a role of EMT in acquired drug resistance to EGFR-TKIs in NSCLC cells with EGFR mutations. By downregulating N-cadherin expression via siRNA, Zhang *et al.* [31] found that the loss of EMT can reduce the proliferation and invasion of erlotinib-resistant H1650ER cells harboring a mutation in exon 19 of the EGFR gene. Vazquez-Martin *et al*. [27] showed that EMT also occurred after PC-9 cells acquired drug resistance to erlotinib. To obtain direct evidence for the role of EMT in acquired resistance to EGFR-TKIs, we firstly induced EMT with TGF-β1 and then evaluated cells' sensitivity to EGFR-TKIs before and after EMT in PC-9 and H460 cells, which are sensitive to EGFR-TKIs at baseline. We observed a mesenchymal phenotype and decreased sensitivity to EGFR-TKIs after EMT induction. We then reversed EMT of PC-9/GR and H460/ER cells by overexpressing E-cadherin and studied their sensitivity to EGFR-TKIs. Reversal of EMT-related morphological and transcriptional features by E-cadherin overexpression in PC-9/GR and H460/ER cells was sufficient to recover cells' sensitivity to gefitinib or erlotinib. These data suggested that EMT, independent of EGFR mutation and MET amplification, is a novel mechanism of EGFR-TKIs-resistance.

Previous studies have shown that IGF-1/IGF1R activation can promote EMT in prostate cancer cells [15] and human mammary epithelial cells [32]. Nurwidya *et al*. [33] found that inhibition of IGF1R reversed hypoxia-induced EMT. We further investigated whether one of the underlying mechanisms of IGF1R on acquired drug resistance is through EMT. We found that along with IGF1R upregulation, both PC-9/GR and H460/ER cells acquired EMT phenotype. Exogenous IGF-1 administration in EGFR-TKIs-naïve PC-9 and H460 cells also triggered IGF1R activation, and subsequently EMT. Furthermore, we showed that siIGF1R in PC-9/GR and H460/ER cells reversed EMT-related morphological and transcriptional features, restored cells' sensitivity to gefitinib or erlotinib. These data suggest IGF1R plays an important role in acquired drug resistance against EGFR-TKIs by inducing EMT.

The mechanism of IGF1R-induced EMT remained unclear in current literatures. Loss of E-cadherin is a critical step in EMT and corresponds with the morphological and cellular alterations [34, 35]. Factors that inhibit E-cadherin expression, such as nuclear transcription factor Snail, ZEB, Slug etc [36] are involved in EMT process. Internalization of the protein complex leads to the degradation of E-cadherin and the relocalization of β-catenin into the nucleus also contributes to this process [37–39]. Data of the present study showed that the expression of Snail was significantly increased after cells acquired drug resistance, and was significantly decreased when IGF1R was silenced, indicating that Snail may be the major transcription factors involved in EMT. Our results also showed that β-catenin relocated from cell membrane into nucleus after IGF-1 induction in NSCLS cells, whereas β-catenin decreased when IGF1R was silenced. Graham *et al.* [15] showed that IGF-1 up-regulates ZEB1 expression through the ERK/MAPK pathway in prostate cancer cells. We herein showed that activation of IGF1R by IGF-1 induced activation of the pERK, but not of pAKT in both PC-9 and H460 cells, whereas pERK decreased dramatically when IGF1R was silenced. Our results indicated that IGF1R may induce EMT in NSCLC cells by upregulating the expression of Snail through activating ERK/AKT pathway, and promoting β-catenin translocation away from cytoplasmic membrane into the nucleus, which directly, repress the expression of E-cadherin.

Taken together, our findings highlight the importance of EMT in IGF1R-induced resistance to EGFR-TKIs in NSCLC. Interference of EMT or IGF1R might be a promising therapeutic strategy for advanced NSCLC.

## MATERIALS AND METHODS

### Cell preparation

Human lung cancer PC-9 cells (mutant EGFR) and H460 cells (wild-type EGFR) were provided by Shanghai Institute Cell Bank of the Chinese Academy of Sciences. Both of the lung cancer cells were cultured in RPMI-1640 with 10% fetal bovine serum (FBS) in a 37°C incubator under 5% CO_2_. Drug resistant PC-9 and H460 cells was obtained by continuous culturing the cells in gefitinib (2.5 μmol/L) or erlotinib (10 μmol/L), each for six months. The resistant PC-9 and H460 cells were designated as PC-9/GR and H460/ER cells, accordingly.

### DNA extraction and detection of EGFR gene mutations

Lung cancer cells in the logarithmic growth phase were trypsinized and collected. DNA extraction was performed using QIAamp DNA Mini Kit (Qiagen, Germany), according to the instruction manual. The integrity of the isolated DNA was assessed by 1.2% agarose gel electrophoresis. EGFR mutations were detected using quantitative PCR-high-resolution melting (qPCR-HRM) curve analysis technology [23].

### C-Met Fluorescence *in situ* hybridization

MET fluorescence *in situ* hybridization (FISH) was performed on unstained formalin-fixed lung cancer cell suspension, using a MET/CEP7 probe cocktail (Kreatech Diagnostics, Amsterdam, Netherlands) according to manufacturer's instructions [24]. A MET/CEP ratio was established on the basis of a count of at least 200 cells. Specifically, two independent observers scored at least 100 non-overlapping interphase nuclei to determine the number of MET gene-specific (red) and CEP7-specific (green) signals. Samples with a ratio of MET/CEP7 greater than 2 were considered to have MET amplification (low amplification < 2, high amplification ≥ 2).

### Cell proliferation assay with MTT

Cells in the logarithmic growth phase were inoculated into 96-well culture plates at 1,500 cells per well. After adherent growth was observed, 100 μL of culture medium (0.5% FBS) containing different concentrations of gefitinib and erlotinib were added to the cells. After 72 h incubation, 20 μL of MTT (5g/L) were added to each well, and incubated for an additional 4 h. The supernatant was then discarded, and 150 μL of DMSO were added. The plates were then agitated for 10 min and the absorbance value (D) were measured at 492 nm with a microplate reader. Cell survival rates after drug treatment and the half maximal inhibitory concentration (IC50) were calculated. Each treatment included six replicates, and three independent experiments were performed.

### Western blotting

Cells from each group were detached with trypsin, centrifuged, and washed 3 times with pre-chilled PBS. Cell lysis buffer (Cell Signaling), containing protease and phosphatase inhibitors, was subsequently added and incubated on ice for protein extraction. Nuclear β-catenin was extracted using NE-PER Nuclear and Cytoplasmic Extraction Reagents (Thermo Scientific). Protein concentration was determined using the BCA Bradford protein assay (Bio-Rad). Equal amounts of proteins were separated via 10% SDS-PAGE and then transferred to a membrane (Amersham). The membrane was blocked in 5% bovine serum albumin (BSA) for 2 h and incubated with an appropriate amount of primary antibody (working dilutions of antibodies: Vimentin 1:100, E-cadherin 1:1,000, Snail 1:2,000, EGFR 1:400, p-EGFR 1:1,000, IGF1R 1:2,000, p-IGF-1R 1:1,000, ERK 1:2,000, p-ERK 1:2,000, AKT1 1:2,000, p-AKT1 1:200, c-MET 1:1000 and β-actin 1:400) (Amersham) in a shaker at 4°C overnight. Detection was by horseradish peroxidase-conjugated secondary antibodies and chemiluminescence. An integrated optical density analysis was performed using Quantity One imaging analysis software.

### Immunofluorescence

Cells were plated into 12-well plates at 2.0 × 10^4^ cells per well. After 48 h, the cells were fixed in 4% paraformaldehyde for 30 min. After washing with PBS for 3 times, the cells were blocked in blocking solution (3% BSA, 0.3% Triton X-100) at room temperature for 60 min. The cells were subsequently incubated with the primary antibodies (rabbit anti-E-cadherin at 1:50; rabbit anti-β-catenin at 1:50; CST) at 4°C overnight. The primary antibodies were then removed, and secondary antibody was added (Cy3-conjugated secondary antibody at 1:500; Life Technologies), and incubated for 1 h. Finally, the nuclei were stained with 0.5 μg/mL DAPI (Sigma), and the fixed cells were observed and photographed using an Olympus IX71 inverted fluorescence microscope (Olympus Optical).

### Scratch test and invasion assay

For Scratch test, cells from each group were inoculated into 6-well plates at 5.0 × 10^4^ cells per well. When cell confluence reached 90%, the cells were starved overnight in serum-free medium. Three parallel lines were scratched on the bottom of the culture plate using a sterile 20 μL micropipette tip. The cells were washed twice using serum-free medium and cultured for another 48 h. The changes in cell motility were observed and photographed under an Olympus inverted fluorescence phase-contrast microscope (40 ×). Three independent experiments were performed.

Cell invasion was determined using Boyden Chamber assay. The top transwell chamber was inoculated with 5 × 10^5^ cells in 200 μL of serum-free culture medium. The bottom chamber was filled with 500 μL of RPMI culture medium containing 10% FBS. After the cells were cultured at 37°C for 24 h, the top chamber was removed, and non-invasive cells remaining in the top chamber were wiped using a cotton swab, followed by crystal violet staining. The cells were placed under an Olympus inverted fluorescence microscope (200 ×) and photographed. Three fields were chosen for counting, and the values were averaged.

### SiIGF1R

According to the sequence information of IGF1R gene (GenBank No., NM-000875), three shRNA was designed using the RNAi design software provided by Ambion. The most efficient interference target sequence “CGA AGA TTT CAC AGT CAA A” was chosen (Supplementary Figure S1). Both strands of the interference sequences were synthesized by Shanghai Genechem. DNA oligo: IGF1-siRNA-a: 5′-CCG GGC CGA AGA TTT CAC AGT CAA ACT CGA GTT TGA CTG TGA AAT CTT CGG CTT TTT G-3′ and IGF1-siRNA-b: 5′-AAT TCA AAA AGC CGA AGA TTT CAC AGT CAA ACT CGA GTT TGA CTG TGA AAT CTT CGG C-3′. Through BLAST homology analysis, no homology with any other genes was found for the selected sequences. In addition, a nonsense sequence was synthesized as the negative control sequence. Plasmid DNA with the siRNA and control sequence were purified and used for lentiviral packaging and titer determination. Then, 293T cells in the logarithmic phase were co-transfected with the recombinant pGC-LV plasmid, pHelper 1.0 plasmid, and pHelper 2.0 plasmid using Lipofectamine 2000 (Invitrogen). After 8 h of transfection, the culture medium was replaced with complete culture medium. Following culture for another 48 h, the supernatant with enriched lentiviral particles was collected. The supernatant was concentrated to obtain a high-titer lentiviral concentrate. PC-9/GR and H460/ER cells in the logarithmic phase were trypsinized to obtain cell suspension and were inoculated into 6-well plates. When cell confluence reached approximately 30%, an appropriate amount of virus was added at a multiplicity of infection (MOI) of 100. Puromycin (5 μg/mL) was added 48 h later. The cells were photographed under a fluorescence microscope to observe the infection rate 72 hours later. A negative control group transfected with lentivirus expressing the empty IGF1R vector (siControl) and a gene silencing group transfected with effective lentivirus expressing IGF1R (siIGF1R) were included in the experiment.

### Stable LV-CDH1 (E-cadherin) gene overexpression

The CDH1(5386–1)-P1 and CDH1(5386–1)-P2 primers, containing exchange pairing bases, enzymatic cutting sites, and a partial 5′-terminal target gene sequence, were designed, synthesized, and then used to amplify the target gene. The sequences of the primers were as follows: CDH1(5386–1)-P1: GAG GAT CCC CGG GTA CCG GTC GCC ACC ATG GGC CCT TGG AGC CGC AG and CDH1(5386–1)-P2: TCA CCA TGG TGG CGA CCG GGT CGT CCT CGC CGC CTC CGT ACA. The amplification products were digested with enzymes and ligated into the lentiviral expression vector GV218. Following transformation, positive clones were picked, and plasmids were extracted after the CDH1 (5386–1) sequence was confirmed via sequencing. GV218-CDH1 (5386–1) and the empty control GV218 plasmid were obtained and transfected into 293T cells. The cells were then cultured in Opti-MEM culture medium containing 1% FBS for 48–72 h. H460/ER and PC-9/GR cells were infected at an MOI of 100. Lentivirus production and cell infection procedures were the same as described above. Cells with more than 80% fluorescence rate were used for subsequent experiments. A negative control transfected with an empty vector lentivirus (Control) and an overexpression control transfected with effective lentivirus expressing CDH1 (CDH1) were included in the experiment.

### Exogenous IGF-1 and TGF-β1 induction

EGFR-TKIs-naïve lung cancer cells PC-9 and H460 were serum-starved overnight and then treated with fresh RPMI 1640 containing 0.5% FBS and 200 ng/ml IGF-I (Sigma-aldrich) for 24 h or TGF-β1 (Sigma-aldrich, 10 ng/mL) for 72 h before proceeding to downstream experiments.

### Statistical analysis

Statistical analyses were performed using the SPSS 17.0 software. The data are presented as means ± standard deviation (SD). Comparisons between two groups were performed using the student *t*-test, while comparisons among multiple groups were performed using one-way analysis of variance (ANOVA) with post hoc least significant difference (LSD) test. *P* < 0.05 was considered to indicate a statistically significant difference.

## SUPPLEMENTARY FIGURES


