# Supplementary material for: Implication of epithelial-mesenchymal transition in IGF1R-induced resistance to EGFR-TKIs in advanced non-small cell lung cancer
Source: Oncotarget. 2015 Nov 5;6(42):44332–45. doi: 10.18632/oncotarget.6293 (PMC4792560; doi:10.18632/oncotarget.6293)
Supplement: Supplementary file 1 [file oncotarget-06-44332-s001.pdf]

SUPPLEMENTARY FIGURES

A

siRNA target sequence specific to IGF-1R

NO.	Target sequence	GC content (%)
1. IGF1R-RNAi(9971)	CCACGTCGAAGAATCGCAT	52.63%
2. IGF1R-RNAi(9972)	GCCGATGTGTGAGAAGACC	57.89%
3. IGF1R-RNAi(9973)	CGAAGATTTCACAGTCAAA	36.84%

B

The construction of lentiviral vector

NO.		Sequence
1	IGF1R-RNAi(9971)-a	ccggcaCCACGTCGAAGAATCGCATctcgagATGCGATTCTTCGACGTGGtgttttttg
	IGF1R-RNAi(9971)-b	aattcaaaaacaCCACGTCGAAGAATCGCATctcgagATGCGATTCTTCGACGTGGtg
2	IGF1R-RNAi(9972)-a	ccggaaGCCGATGTGTGAGAAGACCctcgagGGTCTTCTCACACATCGGCttttttg
	IGF1R-RNAi(9972)-b	aattcaaaaaaaGCCGATGTGTGAGAAGACCctcgagGGTCTTCTCACACATCGGCtt
3	IGF1R-RNAi(9973)-a	ccgggcCGAAGATTTCACAGTCAAActcgagTTTGACTGTGAAATCTTCGgcttttttg
	IGF1R-RNAi(9973)-b	aattcaaaaagcCGAAGATTTCACAGTCAAActcgagTTTGACTGTGAAATCTTCGgc

C

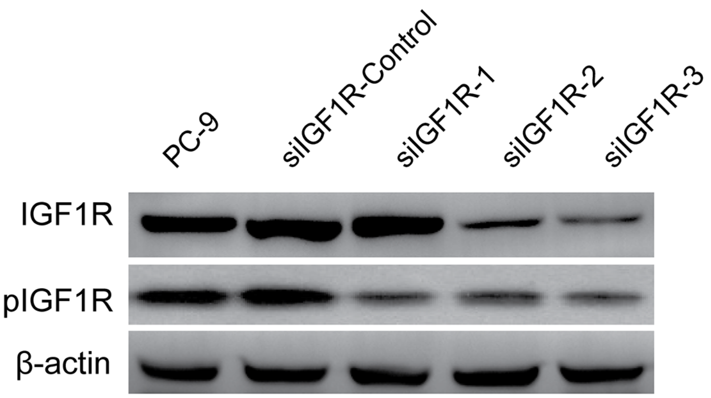

Supplementary Figure S1: Three siRNA was designed with target sequence specific to IGF-1R and the RNA interfering efficiency was tested by immunoblotting analysis. A. Three siRNA target sequence specific to IGF1R designed by the software provided by Ambion. **B.** The construction of lentiviral vector about the three siRNA. **C.** Immunoblotting analysis of IGF1R, phosphor-IGF1R after transfected with siRNA. β-actin was used as an internal control.

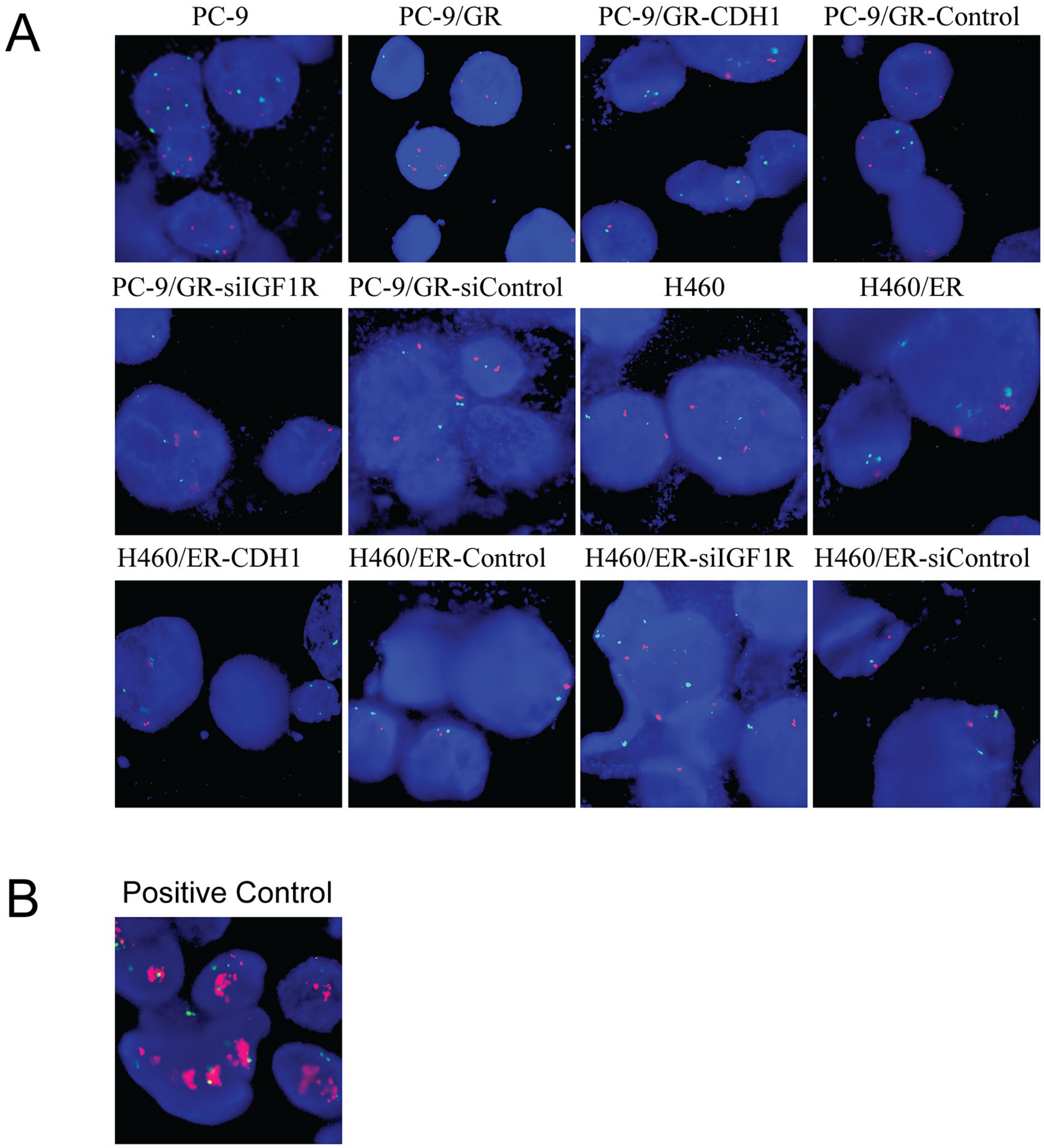

Supplementary Figure S2: FISH assay for detection of c-Met. A. No amplification of the c-Met in lung cancer cells. **B.** c-Met FISH positive case showing a MET/CEP7 ratio ≥ 2. Met gene-specific (red) and CEP7-specific (green) signals and Nuclear DAPI (blue). The photographs were taken at × 400 magnification.

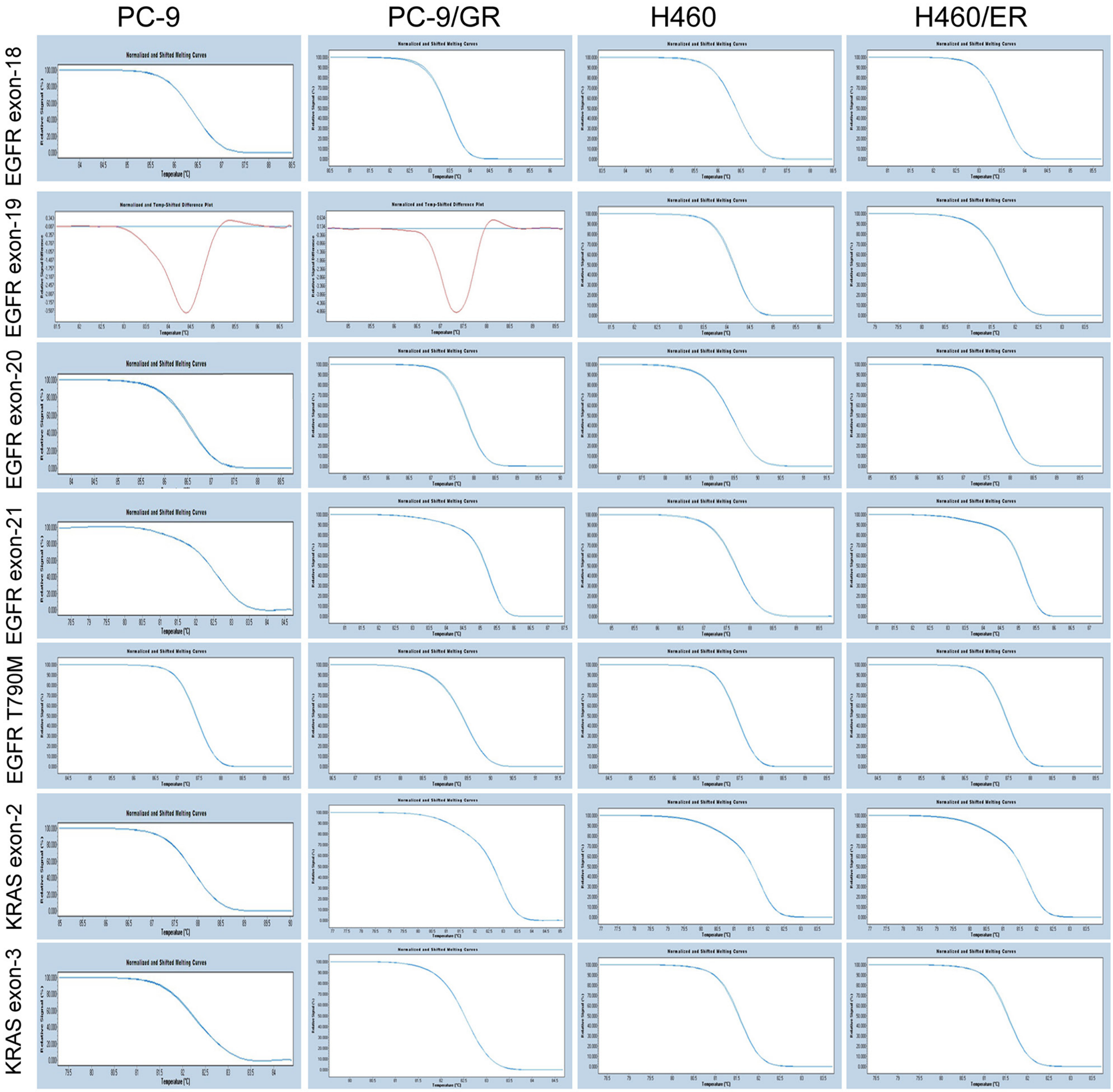

Supplementary Figure S3: Genetic mutations of the EGFR exon 18–21, T790M and KRAS-2,3 in PC-9, H460 cells, and EGFR-TKIs-resistant cells. PC-9 and PC-9/GR had delE746-A750 deletion mutation in exon 19 of EGFR. No EGFR mutation was detected in H460 and H460/ER cells, and all cell lines harbored wild-type KRAS before and after the induction of drug resistance. T790M mutation was not detected in any of the cell lines.

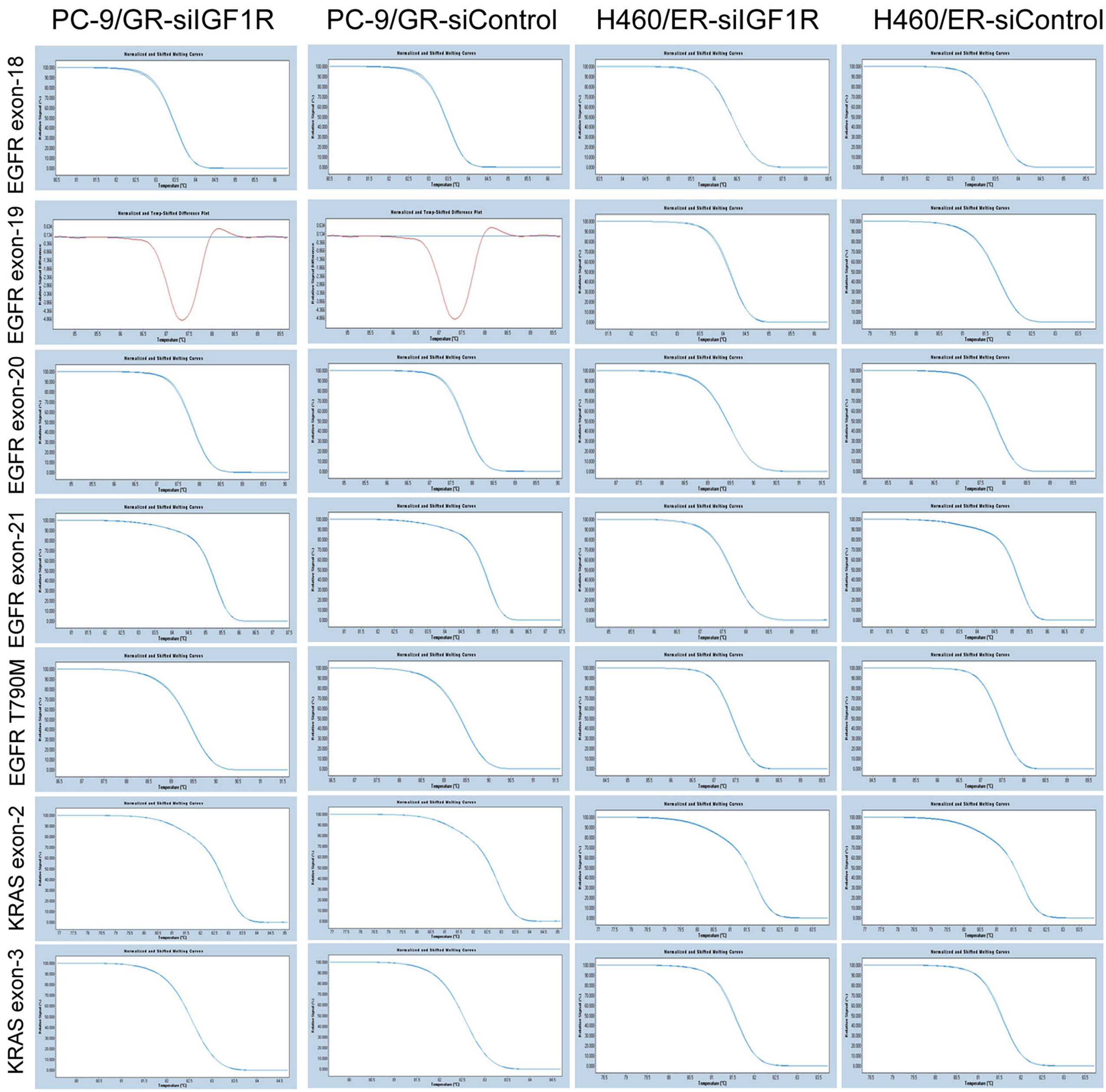

Supplementary Figure S4: Genetic mutations of the EGFR exon 18–21, T790M and KRAS-2,3 in EGFR-TKIs-resistant cells after knocking down IGF1R. PC-9/GR-siIGF1R and PC-9/GR-siControl had delE746-A750 deletion mutation in exon 19 of EGFR. No EGFR mutation was detected in H460/ER-siIGF1R and H460/ER-siControl cells, and all cell lines harbored wild-type KRAS before and after knockdown of IGF1R. T790M mutation was not detected in any of the cell lines.

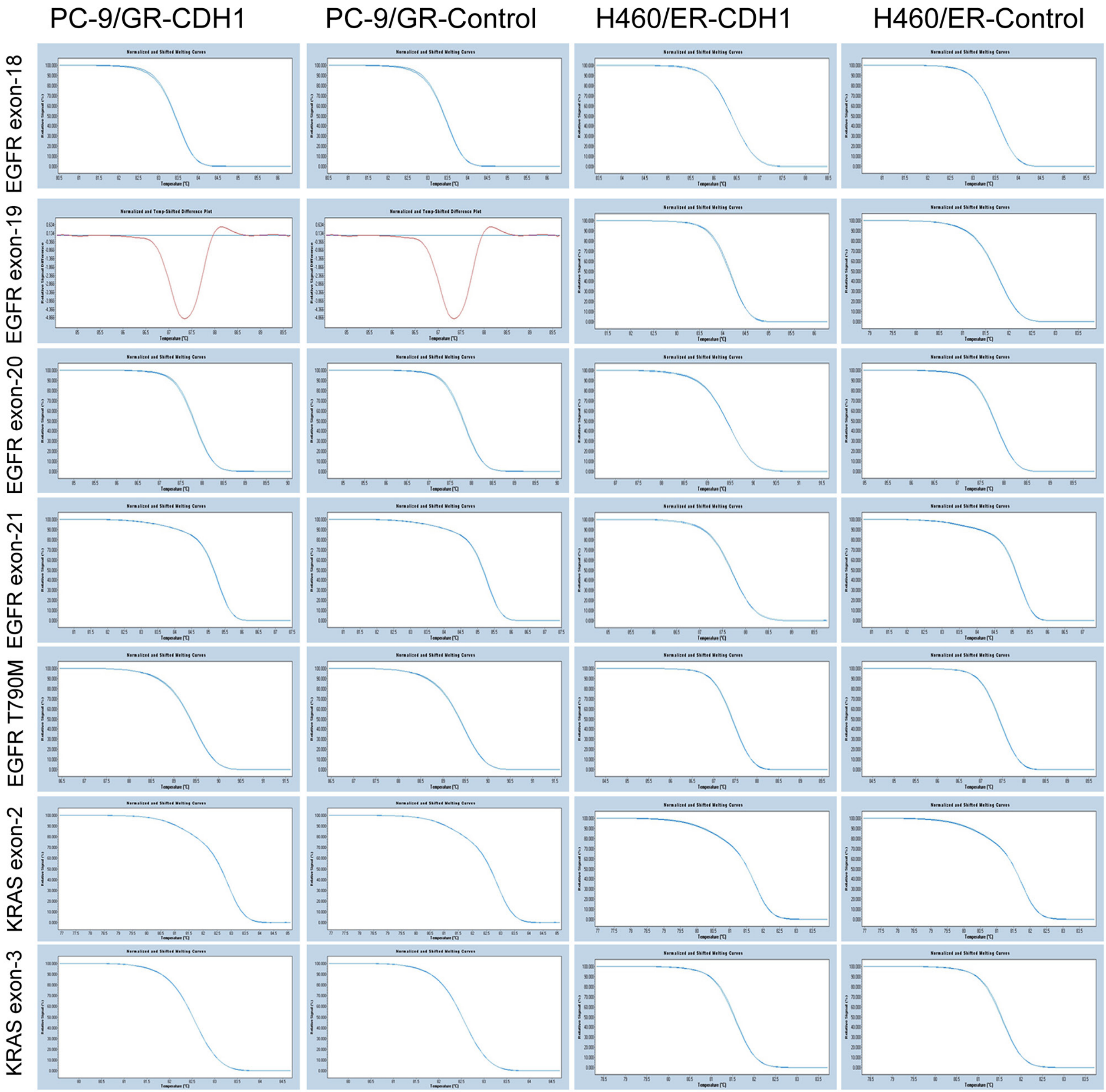

Supplementary Figure S5: Genetic mutations of the EGFR exon 18–21, T790M and KRAS-2,3 in E-cadherin-overexpressing cells. PC-9/GR-CDH1 and PC-9/GR-Control cells had delE746-A750 deletion mutation in exon 19 of EGFR. No EGFR mutation was detected in H460/ER-CDH1 and H460/ER-Control cells, and all cell lines harbored wild-type KRAS before and after E-cadherin overexpression. T790M mutation was not detected in any of the cell lines.

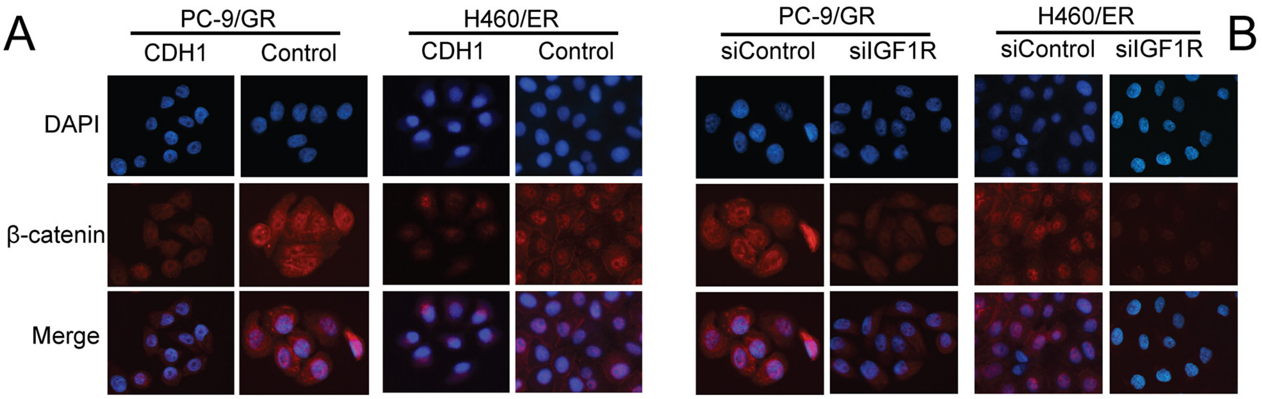

Supplementary Figure S6: Decreased nuclear β-catenin expression after CDH1 overexpression and siIGF1R. Decreased nuclear β-catenin after CDH1 overexpression **A.** and siIGF1R **B.** was confirmed by immunofluorescence staining. The nuclei were stained with DAPI (blue fluorescence), and nuclear β-catenin was stained with Cy3-conjugated antibodies (red fluorescence). The photographs were taken at × 200 magnification.
